# Study on Laser Surface Texturing and Wettability Control of Silicon Nitride Ceramic

**DOI:** 10.3390/mi16070819

**Published:** 2025-07-17

**Authors:** Hong-Jian Wang, Jing-De Huang, Bo Wang, Yang Zhang, Jin Wang

**Affiliations:** 1 School of Intelligent Manufacturing and Aeronautics, Zhuhai College of Science and Technology, Zhuhai 519040, China; 2 School of Electronic Engineering and Intelligentization, Dongguan University of Technology, Dongguan 523808, China

**Keywords:** silicon nitride ceramic, laser surface texturing, wettability control, contact angle

## Abstract

Silicon nitride (Si_3_N_4_) ceramic is widely used in the production of structural components. The surface wettability is closely related to the service life of materials. Laser surface texturing is considered an effective method for controlling surface wettability by processing specific patterns. This research focused on the laser surface texturing of a Si_3_N_4_ ceramic, employing rectangular patterns instead of the typical dimple designs, as these had promising applications in heat transfer and hydrodynamic lubrication. The effects of scanning speed and number of scans on the change of the morphologies and dimensions of the grooves were investigated. The results indicated that the higher scanning speed and fewer number of scans resulted in less damage to the textured surface. As the scanning speed increased, the width and depth of the grooves decreased significantly first, and then fluctuated. Conversely, increasing the number of scans led to an increase in the width and depth of the grooves, eventually stabilizing. The analysis of the elemental composition of different areas on the textured surface presented a notable increase in oxygen content at the grooves, while Si and N levels decreased. It was mainly caused by the chemical reaction between Si_3_N_4_ ceramic and oxygen during laser surface texturing in an air environment. This study also assessed the wettability of the textured surface, finding that the contact angle of the water droplet was significantly affected by the groove dimensions. After laser surface texturing, the contact angle increased from 35.51 ± 0.33° to 57.52 ± 1.83°. Improved wettability was associated with smaller groove volume, indicating better hydrophilicity at lower scanning speed and enhanced hydrophobicity with a fewer number of scans.

## 1. Introduction

Silicon nitride (Si_3_N_4_) ceramic is extensively utilized in industry due to its outstanding mechanical properties, biocompatibility and excellent corrosion resistance [1,2,3]. As an important structural material, Si_3_N_4_ ceramic is commonly used to produce components like cutting tools [4], bearing balls [5], and artificial joints [6]. The durability of these components is closely linked to the lifespan of the materials. In engineering scenarios, component failures are often attributed to wear caused by complex operating conditions [7]. To enhance the contact conditions between materials and prolong their lifespan, lubricants are typically employed. Wettability control has emerged as an effective and convenient method to reduce damage by altering the effects of lubrication [8,9,10], primarily achieved through the surface modification of the materials. However, the hard and brittle nature of Si_3_N_4_ ceramic poses challenges for modifying the working area using mechanical processing techniques. While the common way is chemical treatment [11], it is not ideal for ceramic components that experience frequent relative motion. Laser surface texturing, on the other hand, is well-suited for ceramics and is considered an optimal approach for achieving wettability control by creating patterns on the surface of materials [12,13,14].

Wettability is typically assessed by measuring the contact angle of a liquid droplet on the surface of materials [15]. Recent research has primarily concentrated on oxide ceramics. Yan et al. [16] utilized laser surface texturing to create arc grooves and square pillar arrays on zirconia ceramic, resulting in a contact angle variation from 10° to 133°. The surface exhibited both superhydrophilic and hydrophobic characteristics. Pu et al. [17] combined laser surface texturing with silanization to manipulate the wettability of zirconia ceramic, enhancing its hydrophobicity through alterations in chemical bonds. Although laser surface texturing induced phase transitions on the surface of the materials, the contact angle was not obviously affected [18]. Sun et al. [19] applied a femtosecond laser to regulate the surface wettability of yttria-stabilized zirconia ceramic. This study achieved the transition of the surface from hydrophilic to superhydrophobic. Zheng et al. [20] used a femtosecond laser to achieve reversible wettability on the surface of zirconia ceramic. This alteration in the wettability was mainly caused by the rapid adsorption of airborne organic substances in a heating environment. Ghalandarzadeh et al. [21] compared the wettability of the patterned versus the original surface of zirconia ceramic. The textured surface exhibited superior hydrophobicity, which helped reduce bacterial adhesion. Cao et al. [22] obtained a superhydrophilic surface on alumina ceramic through laser surface texturing with linear grooves. The results indicated that patterns created in an air environment were more effective in enhancing the durability of hydrophilicity compared to those made in an argon environment. Bai et al. [23] conducted laser surface texturing on silicon carbide ceramic. The textured surface of the material was superhydrophilic due to the increased thermal residual stress. Mukhopadhyay et al. [24] explored wettability control on the surface of aluminum nitride ceramic, revealing that the superhydrophobic surface could be beneficial for the liquid cooling of electronic components.

As for Si_3_N_4_ ceramic, Yang et al. [25] examined the impacts of dimples on the wettability. They found that as laser power and scanning passes increased, the dimensions of the dimples also increased. The contact angle initially decreased with rising laser power but then increased, although it continued to drop with additional scanning passes. Further research indicated that a higher overlap rate of laser pulses enhanced hydrophilicity [26]. Wang et al. [27] manipulated the wettability of Si_3_N_4_ ceramic through laser surface texturing using various patterns, discovering that triangular patterns resulted in a lower contact angle and improved hydrophilicity compared to hexagonal patterns. While dimples are the most frequently utilized patterns for laser surface texturing of Si_3_N_4_ ceramic [28,29], there is limited research on using other patterns for wettability control. In recent years, rectangular patterns present advantages in functional surface applications, particularly in heat transfer [30] and hydrodynamic lubrication [31]. Moreover, the scale of rectangular patterns is greater than that of micro dimples, which simplifies the processing [32]. This study employed different laser processing parameters to create rectangular patterns on Si_3_N_4_ ceramic, investigating how these parameters affected the dimensions and morphologies of textured patterns. The contact angle of the Si_3_N_4_ ceramic was measured, and the wetting mechanism of the textured patterns was discussed. This paper serves as a reference for controlling the wettability of structural ceramics through laser surface texturing.

## 2. Materials and Methods

**Figure 1:**
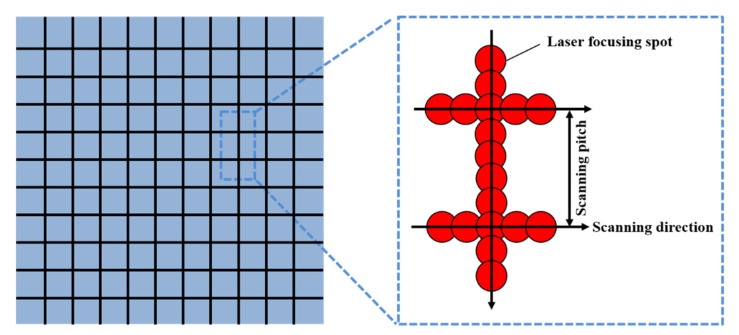
The schematic diagram of laser surface texturing of Si_3_N_4_ ceramic.

The testing material was bulk Si_3_N_4_ ceramic (Shenzhen Hard Precision Ceramic Co., Ltd., Shenzhen, China). The schematic diagram illustrating the laser surface texturing of the Si_3_N_4_ ceramic is presented in Figure 1. Prior to the laser treatment, the surface of materials was mirror-polished. The texturing procedure was performed using a picosecond laser (RA-RIR-60, Jiangsu Raytolaser Technology Co., Ltd., Suqian, China) with a pulse width of 10 ps and a wavelength of 1064 nm. The diameter of focused spot on the surface of Si_3_N_4_ ceramic was approximately 20 μm. The laser power was operated at a power of 9 W, with a repetition frequency of 60 kHz and a scanning pitch of 300 μm. Various scanning speeds (60 mm/s, 120 mm/s, 180 mm/s, 240 mm/s, 300 mm/s) and the number of scans (5, 15, 25, 35, 45) were used as processing variables. Before and after the laser surface texturing and subsequent characterization, Si_3_N_4_ ceramic was ultrasonically cleaned in ethanol for 20 min to remove contaminants and debris.

The measurement of contact angle on the Si_3_N_4_ ceramic was performed using a wettability testing system (SDC-350H, Shengding Precision Instrument, Dongguan, China) employing the sessile drop method. A water droplet of 2 μL was produced by a liquid purification system (Unique-R30, RSJ Scientific Instruments, Xiamen, China). For the textured surfaces obtained under each processing parameter, the contact angle was measured three times. The groove dimensions were examined by the optical microscope (DSX510, Olympus, Tokyo, Japan). The surface morphology of the textured patterns was examined by scanning electron microscopy (SEM, Gemini-360, Zeiss, Oberkochen, Germany). The elemental composition was characterized by energy-dispersive spectroscopy (EDS) integrated with SEM.

## 3. Results and Discussion

### 3.1. Laser Surface Texturing of Si_3_N_4_ Ceramic with Different Scanning Speeds

**Figure 2:**
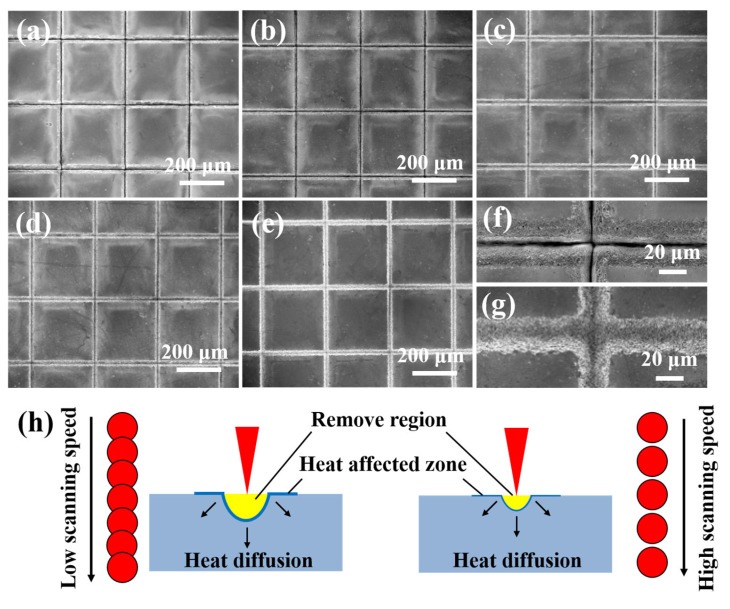
SEM of morphology on textured surface of Si_3_N_4_ ceramic with different scanning speeds: (**a**) 60 mm/s, (**b**) 120 mm/s, (**c**) 180 mm/s, (**d**) 240 mm/s, (**e**) 300 mm/s. (**f**,**g**) are enlarged views of grooves from (**a**) to (**e**), respectively. (**h**) Schematic diagram of processing groove at low and high scanning speeds (The number of scans: 25).

Figure 2a–f shows the SEM images of the textured surface of the Si_3_N_4_ ceramic at various scanning speeds. The surface exhibited distinct rectangular patterns, with noticeable grooves between each pattern. At a scanning speed of 60 mm/s (Figure 2a), the bottoms of the grooves were not easily visible due to the processing parameters. As the scanning speed increased, the duration of the laser’s exposure per unit length of the grooves decreased, resulting in lower input linear energy [33]. Consequently, the bottoms of the grooves became more apparent (Figure 2b–e), indicating that the depth of the textured patterns was reduced. This observation was further supported by comparing the morphology of the grooves. While the rectangular patterns appeared similar, there were morphological differences. At lower scanning speeds, the edges of the grooves exhibited significant damage and irregularity (Figure 2f), primarily due to the spatial characteristics of the laser energy. The groove had a peaked shape due to the Gaussian energy distribution, resembling the taper seen in laser drilling of materials [34,35]. The energy at the groove edges was less intense than in the center, and repeated scanning resulted in non-straight edges. In contrast, at higher scanning speed, the groove shape was more linear and uniform (Figure 2g). The laser’s shorter exposure time at repeated processing points reduced the impact of laser energy on the groove edges. The bottoms of the grooves were clearly visible compared to the narrow slits formed at a lower scanning speed. It also indicated that the increase in the scanning speed enhanced the regularity of the grooves. A schematic diagram of the processing groove at both low and high scanning speeds is presented in Figure 2h. The molten layer is not drawn. Obviously, the overlap rate of the laser-focused spot varies with the scanning speed. This directly leads to a linear energy difference in the laser input along the scanning path. Normally, more materials are removed at a slower scanning speed, but this also causes greater thermal impact on the processing area. Although thermal effects can be effectively reduced by increasing the scanning speed, the amounts of material removed are reduced.

**Figure 3:**
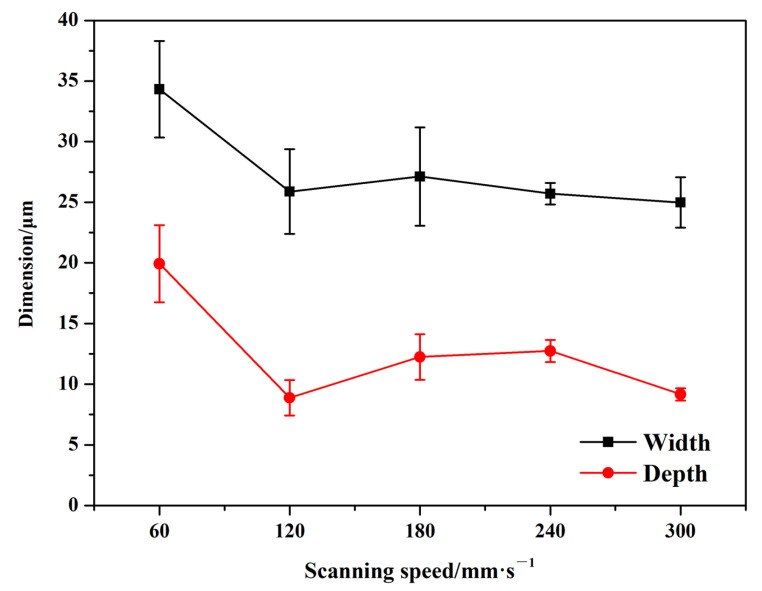
Dimensions of textured patterns on Si_3_N_4_ ceramic surface with different scanning speeds (The number of scans: 25).

Figure 3 illustrates the dimensions of textured patterns on the surface of the Si_3_N_4_ ceramic at different scanning speeds. As the scanning speed increased from 60 mm/s to 120 mm/s, there was a notable reduction in both the width and depth of the grooves. This trend showed that the textured patterns became narrower and shallower. The process of laser surface texturing involves the formation and removal of slags. Although a greater amount of slag was produced at lower scanning speeds, the higher linear energy facilitated the removal of materials more easily. Conversely, as the scanning speed increased, material removal became more difficult due to the reduced linear energy impacting the grooves, resulting in smaller groove dimensions. It is important to note that at higher scanning speeds, there was also a decrease in slag formation during the laser surface texturing. When the scanning speed further increased from 120 mm/s to 180 mm/s, the dimensions of the groove patterns experienced a slight increase. This change might be attributed to a reduction in laser spot overlap within the processing parameters, leading to less slag formation and more effective removal of materials. When the scanning speed increased from 180 mm/s to 300 mm/s, the dimensions of the grooves remained relatively stable, with width fluctuations being less pronounced than those in depth. It indicated that the width was less affected by changes in processing parameters compared to the depth of the grooves. This phenomenon is also observed in the laser grooving of materials [36].

### 3.2. Laser Surface Texturing of Si_3_N_4_ Ceramic with Different Numbers of Scans

**Figure 4:**
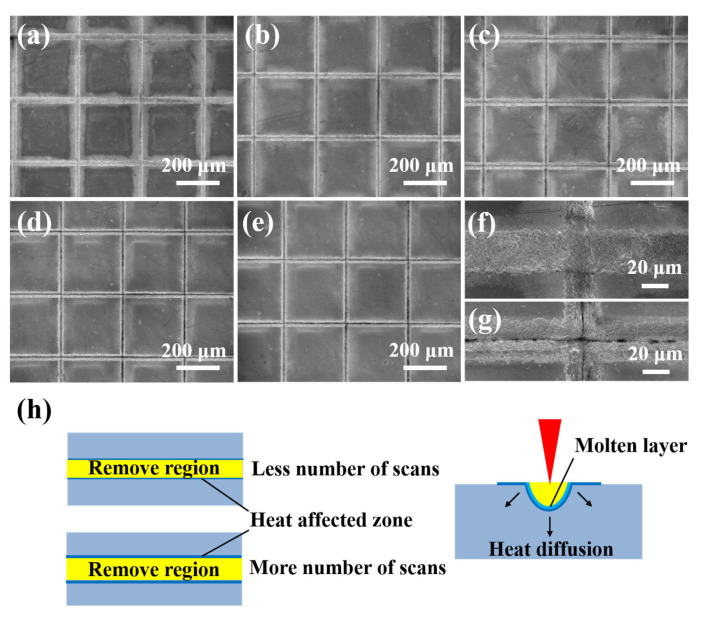
SEM of morphology on textured surface of Si_3_N_4_ ceramic with different number of scans: (**a**) 5, (**b**) 15, (**c**) 25, (**d**) 35, (**e**) 45. (**f**,**g**) are enlarged views of grooves from (**a**) to (**e**), respectively. (**h**) Schematic diagram of processing groove with fewer and greater numbers of scans (The scanning speed: 180 mm/s).

Figure 4a–g presents SEM images of the morphology on the textured surface of Si_3_N_4_ ceramic with different numbers of scans. The change in the number of scans was achieved by altering the processing times along the same path to create grooves on the surface of the materials. Notably, the grooves were shallowest with the number of scans at five (Figure 4a), allowing for clear visibility of the bottom of the textured patterns. As the number of scans increased to 15, the grooves underwent repeated processing, yet the overall structure remained discernible (Figure 4b). Generally, the removal of Si_3_N_4_ ceramic increased with more scans. It meant that a higher number of scans resulted in clearer textured patterns. However, the morphology of the textured patterns remained similar when the number of scans ranged from 25 to 45 (Figure 4c–e). This phenomenon might be attributed to the accumulation of slags in the processing area, which settled at the bottom of the grooves and were repeatedly heated by the laser beam. These residues obstructed the laser energy transmission to the unprocessed regions. A comparison of the enlarged views of grooves with the number of scans at five (Figure 4f) and 45 (Figure 4g) revealed significant differences in the textured patterns. The grooves from the former sample were noticeably smoother and more continuous than those from the latter sample, where the direct laser action on the molten materials led to the discontinuity of grooves at higher scanning times. Figure 4h exhibits a schematic diagram of the processing groove at both a smaller and a greater number of scans. More materials are removed at more scanning times and the heat-affected zone is also larger. However, with more scanning passes, the same area is treated multiple times, leading to the creation of a thicker molten layer. Therefore, the grooves processed with a higher number of scans appear discontinuous.

**Figure 5:**
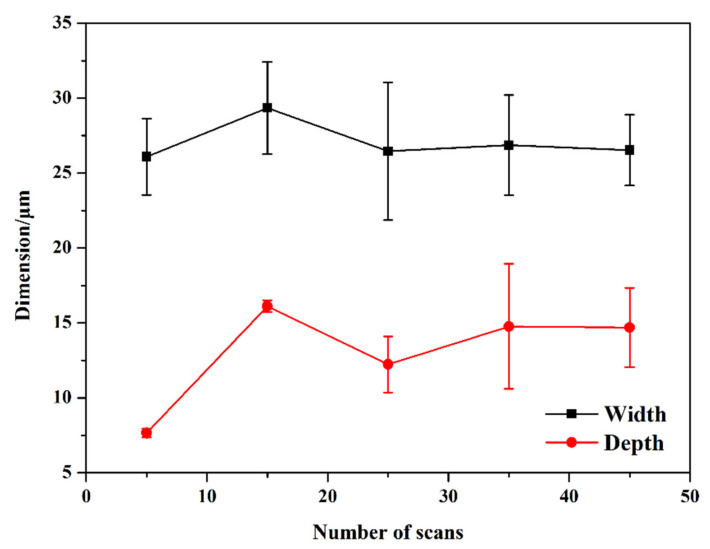
Dimensions of textured patterns on Si_3_N_4_ ceramic surface with different numbers of scans (The scanning speed: 180 mm/s).

Figure 5 shows the dimensions of textured patterns on the surface of Si_3_N_4_ ceramic with a varying number of scans. The depth of the grooves presented more significant changes with the number of scans compared to the width. The main reason might be that the energy from the laser beam was primarily focused on the depth direction of the grooves. As a result, multiple scans had a greater impact on the depth, leading to more pronounced changes. This phenomenon was also observed in the groove processing of Ni alloy [37] and glass substrate [38]. The effects of increasing the number of scans and the scanning speed on the textured patterns differed. As the number of scans increased from 5 to 25, both the width and depth of the groove increased initially, and then decreased slightly. This behavior contrasted with the slight increase in groove dimension observed with higher scanning speed. The increase in scanning times led to more accumulation of slags at the bottom of the grooves. These residues were difficult to remove. Consequently, the dimensions of the grooves did not increase continuously with more scans but instead began to decrease. In other words, processing the grooves became more challenging at greater depths. As the number of scans continued to rise, the dimensions of the textured patterns stabilized, indicating that the laser surface texturing had reached a balanced state.

### 3.3. Morphology and EDS Analysis on the Textured Surface of Si_3_N_4_ Ceramic

**Figure 6:**
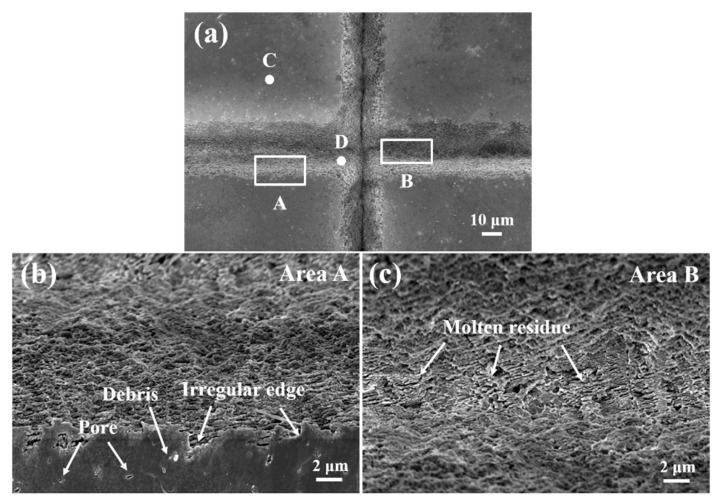
SEM of morphology on textured surface (**a**) and enlarged views at edge (**b**), bottom (**c**) of grooves (The scanning speed and number of scans: 180 mm/s, 25).

**Figure 7:**
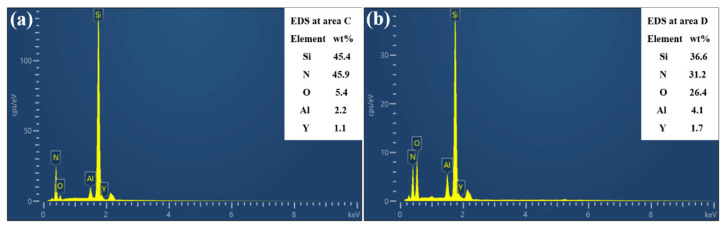
EDS analysis at surface (**a**) and bottom (**b**) of textured patterns in Figure 6.

Figure 6 displays the SEM images of the morphology of the textured grooves of Si_3_N_4_ ceramic. It can be seen that the horizontal and vertical grooves were evenly distributed (Figure 6a). Despite the laser’s scanning path being straight, the edges of the grooves in the textured patterns appeared irregular (Figure 6b). During the laser surface texturing process, the laser energy was directed from the surface of the materials into its depth. As the textured patterns formed, materials were continuously removed from the bottom of the grooves. The laser beam repeatedly scanned the edges of the grooves, resulting in a textured surface created through multiple heating and cooling cycles. This process also involved the thermal expansion of internal pores and the ejection of processing debris, contributing to the irregularity of the groove edges. In contrast, the morphology at the bottom of the grooves was distinct (Figure 6c). Since the grooves were created by processing the materials, it was necessary to extract the Si_3_N_4_ ceramic from the groove bottoms. Unlike laser through-hole machining [39], the laser textured patterns were classified as blind groove structures, which meant that the formed slags could not be completely removed during laser surface texturing. Consequently, molten residues accumulated at the bottom of the grooves, obstructing further processing and complicating the manufacturing of textured patterns. Figure 7 presents EDS analysis of various areas within the textured patterns shown in Figure 6. Area C, located on the rectangular structure of the textured patterns, primarily contained Si and N (Figure 7a). It was consistent with the composition of the Si_3_N_4_ ceramic. During the preparation of the materials, oxides like Al_2_O_3_ and Y_2_O_3_ were often added as sintering aids. Therefore, elements of O, Al and Y were detected. The elemental composition at area D differed significantly from that at area C (Figure 7b), with a notable decrease in Si and N content and a marked increase in O content. This change could be attributed to the decomposition of Si_3_N_4_ ceramic during the laser surface texturing in an air environment, where Si reacted with O to form silicon oxide [40]. It was also the main component of the molten residues at the bottom of the grooves. With the lower scanning speed and fewer number of scans, the oxidation of materials was more severe, resulting in higher content of SiOx, which made the better hydrophilicity on the textured surface [41]. This was validated in a later chapter.

### 3.4. Wetting State and Contact Angle on Si_3_N_4_ Ceramic with Different Texturing Parameters

**Figure 8:**
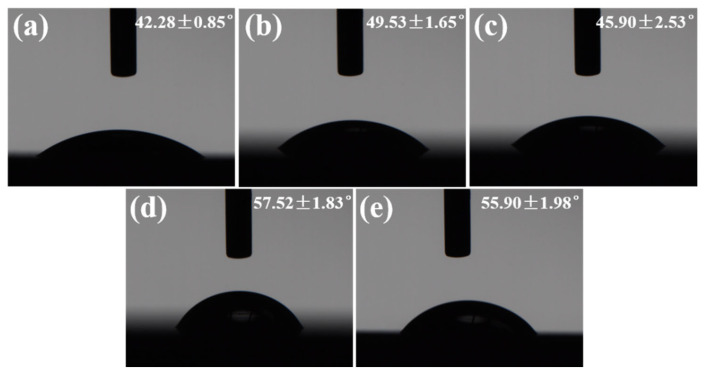
The wetting state of water droplet on the textured surface of Si_3_N_4_ ceramic with different scanning speeds: (**a**) 60 mm/s, (**b**) 120 mm/s, (**c**) 180 mm/s, (**d**) 240 mm/s, (**e**) 300 mm/s (The number of scans: 25).

**Figure 9:**
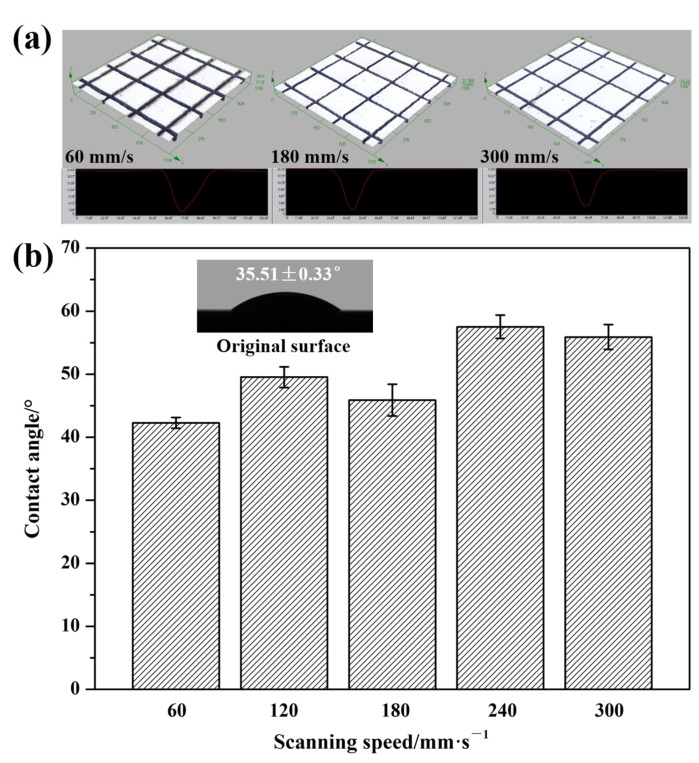
The 3D surface reconstructions of the grooves (**a**) and the contact angle of water droplet on the textured surface of Si_3_N_4_ ceramic with different scanning speeds (**b**) (The number of scans: 25).

Figure 8 illustrates the wetting state of a water droplet on the textured surface of Si_3_N_4_ ceramic at various scanning speeds. The water droplet on the surface of the material was not spherical. Instead, it adopted a spherical crown shape, indicating that the water droplet spread across the contact surface. This observation suggested that the textured surface exhibited hydrophilic properties. Notably, the contact area between the water droplet and the surface of the materials, as well as the height of the water droplet, were different. The results indicated that there were differences in the wettability of the textured surface. Figure 9 presents the 3D surface reconstructions of the grooves and contact angle of the water droplet on the textured surface of the Si_3_N_4_ ceramic as the scanning speed ranged from 60 mm/s to 300 mm/s. It can be visually observed that the dimensions of the grooves decreased with an increase in the scanning speed (Figure 9a). The groove patterns had a larger structural scale than LIPSS patterns on the surface of materials [42]. As the scanning speed increased, the contact angle showed a fluctuating upward trend, implying a reduction in the surface’s hydrophilicity at higher scanning speed (Figure 9b). The contact angle of the original surface was 35.51 ± 0.33°, indicating better hydrophilicity. Wettability was closely linked to the microstructure of the surface of materials [43]. The introduction of textured patterns reduced the contact area between the water droplet and the material surface, and increased the contact angle. As previously mentioned, the dimensions of the textured patterns decreased with increasing scanning speed. The trend in variation of the dimensions led to a reduction in groove volume. Consequently, it became easier for air to be trapped in the grooves when the water droplet made contact with the textured surface. This was beneficial for increasing the contact angle of the water droplet. By changing the scanning speed, the variation range of the contact angle reached about 20°. Compared to parallel line patterns [44,45], the textured surface with rectangular patterns had no wetting direction selectivity and better uniformity. Additionally, better surface integrity was achieved with lower laser energy input at a higher scanning speed. This change in processing parameters also influenced the wettability of the textured surface.

**Figure 10:**
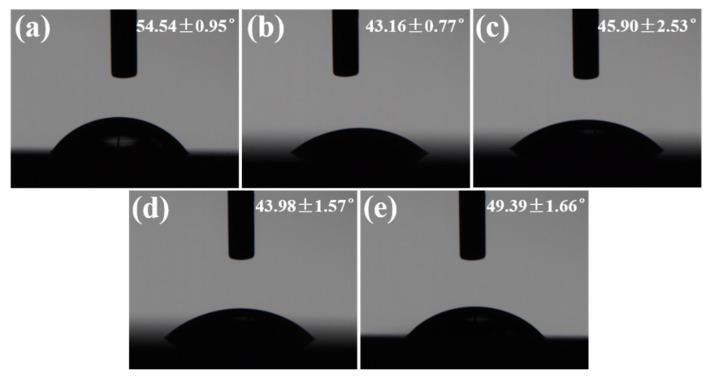
The wetting state of water droplet on the textured surface of Si_3_N_4_ ceramic with different numbers of scans: (**a**) 5, (**b**) 15, (**c**) 25, (**d**) 35, (**e**) 45 (The scanning speed: 180 mm/s).

**Figure 11:**
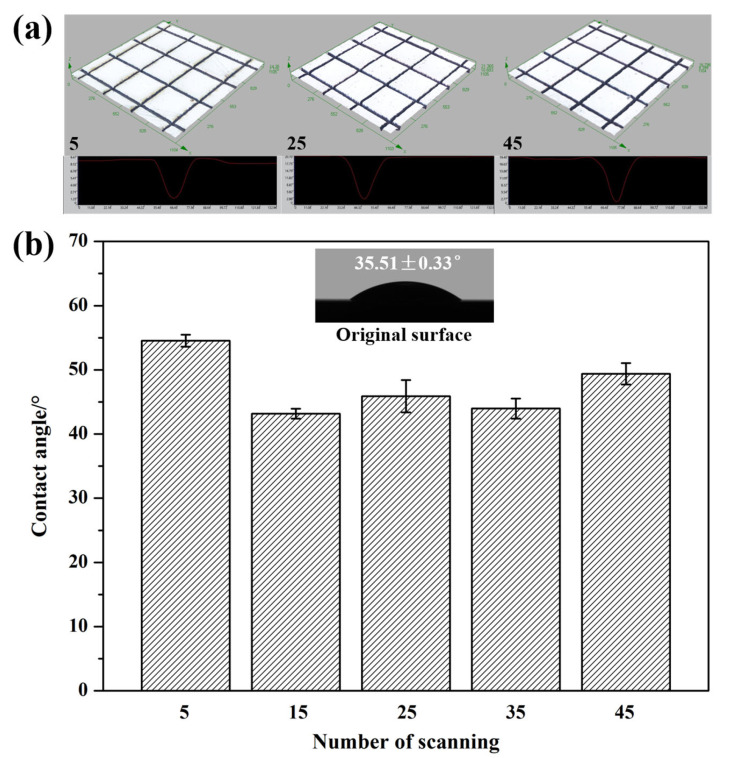
The 3D surface reconstructions of the grooves (**a**) and the contact angle of water droplet on the textured surface of Si_3_N_4_ ceramic with different numbers of scans (**b**) (The scanning speed: 180 mm/s).

Figure 10 shows the wetting state of a water droplet on the textured surface of Si_3_N_4_ ceramic with different numbers of scans. Although the water droplet still spread across the surface of the materials as the number of scans increased, the change in wettability contrasted with the trend of increasing scanning speed. This phenomenon could be attributed to alterations in the dimensions of the textured patterns based on processing parameters. Unlike the scanning speed, the dimensions of the grooves increased with more scanning times, which in turn increased the volume of the grooves. This made it easier for the water droplet to penetrate the grooves, enhancing the wettability of the textured surface. Figure 11 presents the 3D surface reconstructions of the grooves and the contact angle of the water droplet on the textured surface of Si_3_N_4_ ceramic for the number of scans ranging from 5 to 45. In contrast to increasing the scanning speed, the dimensions of the grooves increased with the number of scans (Figure 11a). When the number of scans was five, the contact angle was over 50° (Figure 11b). As the scanning times increased, the contact angle significantly decreased and then fluctuated. These results indicated that the wettability of the textured surface was better. Upon contact, the water droplet spread in various directions on the textured surface while also penetrating into the grooves. This phenomenon did not exist on the original surface. The increase in scanning time from 5 to 15 resulted in a notable change in groove depth, which directly caused the contact angle to drop. Within this range of processing parameters, the contact angle was more influenced by changes in groove depth than width. As the number of scans continued to rise from 15 to 45, the contact angle stabilized due to the minimal changes in the dimensions of the textured patterns. The effects of the number of scans on the contact angle were not as significant as the scanning speed. Overall, the impact of the number of scans on the contact angle of the water droplet was contrary to that of the scanning speed.

## 4. Conclusions

In the current work, laser surface texturing of rectangular patterns was investigated on a Si_3_N_4_ ceramic. The research analyzed how variations in the scanning speed and the number of scans affected the dimensions and morphologies of the textured surface. Additionally, the elemental composition in various areas of the textured patterns was examined. The impact of processing parameters on the wetting state and contact angle of water droplets on the textured surface was also studied. Rectangular patterns were easier to process than dimple patterns, and presented better thermal conductivity and hydrodynamic lubrication characteristics, which made them a promising option for use in micro-devices. The main conclusions are outlined as follows:

1. As the scanning speed increased, the bottoms of the grooves became more apparent. The edges of the grooves appeared more uniform. This was closely linked to how the laser energy was distributed spatially. When the scanning speed increased from 60 mm/s to 120 mm/s, there was a notable reduction in both the width and depth of the grooves. With further increases in the scanning speed, the width and depth of the grooves presented only slight fluctuations around 25 μm and 10 μm, respectively.
2. The impact of the number of scans on the textured patterns was contrary to that of the scanning speed. The grooves reached the minimum width of ~7 μm and depth of ~26 μm when the number of scans was five. As the number of scans increased, the dimensions of the grooves grew rapidly before stabilizing. The accumulation of slags at the bottom of the grooves impeded further processing and led to inconsistencies in the textured areas.
3. The continuous process of heating and cooling the textured regions led to the development of uneven groove edges following several laser scans. Pores and debris were noted around the borders of the textured patterns. Elemental analysis revealed that the rectangular section was primarily composed of Si and N. Conversely, the O levels in the groove increased considerably, while the amounts of Si and N decreased. It was mainly caused by the chemical reactions that took place during the laser surface texturing in an air environment.
4. The scanning speed and the number of scans were important factors that influenced the wettability of the textured surface, but the effects were contrary. The hydrophobicity of material surfaces could be enhanced by laser surface texturing from 35.51 ± 0.33° to 57.52 ± 1.83°. These findings were primarily related to the variations in the dimensions of groove patterns based on different processing parameters. A lower scanning speed was beneficial for improving the hydrophilicity, while a fewer number of scans effectively enhanced the hydrophobicity of the textured surface.

## Data Availability

The original contributions presented in this study are included in the article. Further inquiries can be directed to the corresponding author.
